# Combined Nanofiltration and Thermocatalysis for the Simultaneous Degradation of Micropollutants, Fouling Mitigation and Water Purification

**DOI:** 10.3390/membranes11080639

**Published:** 2021-08-19

**Authors:** Katarzyna Janowska, Xianzheng Ma, Vittorio Boffa, Mads Koustrup Jørgensen, Victor M. Candelario

**Affiliations:** 1 Center for Membrane Technology, Department of Chemistry and Bioscience, Aalborg University, Fredrik Bajers Vej 7H, 9220 Aalborg, Denmark; kaj@bio.aau.dk (K.J.); xm@bio.aau.dk (X.M.); mkj@bio.aau.dk (M.K.J.); 2 Department of Research and Development, LiqTech Ceramics A/S, Industriparken 22C, 2750 Ballerup, Denmark; vcl@liqtech.com

**Keywords:** nanofiltration, thermocatalysis, perovskite, micropollutants, water purification, wastewater

## Abstract

Due to progressive limitation of access to clean drinkable water, it is nowadays a priority to find an effective method of water purification from those emerging organic contaminants, which might have potentially harmful and irreversible effects on living organisms and environment. This manuscript reports the development of a new strategy for water purification, which combines a novel and recently developed Al_2_O_3_-doped silica nanofiltration membrane with a thermocatalytic perovskite, namely cerium-doped strontium ferrate (CSF). The thermocatalytic activity of CSF offers the opportunity to degrade organic pollutants with no light and without input of chemical oxidants, providing simplicity of operation. Moreover, our studies on real samples of secondary effluent from wastewater treatment showed that the thermocatalyst has the ability to degrade also part of the non-toxic organic matter, which allows for reducing the chemical oxygen demand of the retentate and mitigating membrane fouling during filtration. Therefore, the new technology is effective in the production of clean feed and permeate and has a potential to be used in degradation of micropollutants in water treatment.

## 1. Introduction

Sources of global, usable clean drinking water are dramatically decreasing and it is a lifeline to save our planet from a water crisis. Municipal, industrial and agricultural wastewaters are the main sources of micropollutants causing the contamination of natural and drinking water. These compounds such as personal care products, pesticides, pharmaceuticals, hormones, industrial chemicals and environmental estrogens, even at low concentrations (ng L^−1^ or µg L^−1^), have damaging and irreversible effects on living organisms and the environment [1,2]. Therefore, it is a priority to develop an efficient technology to remove these contaminants from wastewater, considering that, in conventional wastewater treatment plants (WWTPs), physical methods are unproductive for their abatement [3], and biological processes can provide only a limited degradation of such pollutants [4,5]. Therefore, a number of advanced wastewater treatment technologies such as activated carbon adsorption, advanced oxidation processes and membrane technologies have been used for water purification [6]. Above all, membrane-based technologies are recently gaining attention as they produce water of superior quality, they are less sensitive to feed quality fluctuations, and their footprint is much smaller compared to conventional water treatment processes [4]. Out of membrane technologies, especially nanofiltration (NF) has been found as a promising cost-effective alternative method for removing and concentrating low-molecular-weight organic micropollutants [7,8]. The pore sizes of NF membranes are typically between 1 and 2 nm, so they can molecularly sieve hydrated multivalent ions and organic micropollutants [9,10]. NF is distinguished by low operating pressure and high permeability, which benefits in the form of relatively low investment, operation and maintenance costs [11]. Nevertheless, there are some limitations of this process such as the non-complete rejection of water pollutants, membrane fouling, and the production of a toxic concentrate (retentate), which needs to be treated before disposal [2,6,8,12,13].

This manuscript reports the development of a novel strategy for water purification that involves the integration of membrane filtration and advanced oxidation. For filtration experiments, we used a ceramic membrane consisting of alumina tubular support coated with an Al_2_O_3_-doped NF silica layer, which has been previously reported [14], showing good retention of organic pollutants. Al_2_O_3_ doping was used to increase the chemical stability of the silica thin layer [14,15]. Moreover, the tubular configuration and the mechanical resistance of this membrane are suitable to perform the NF experiment in the presence of thermocatalytic particles, which might clog or scratch commercial polymeric membrane modules. Advanced oxidation processes (AOPs) utilize highly reactive oxygen species (ROS) such as OH• and O_2_•‒, which mineralize most of the pollutants into less or non-toxic products (e.g., H_2_O and CO_2_) in aqueous systems [3,16,17]. Among AOPs, thermal catalysis offers the opportunity to degrade organic pollutants with no light and without input of chemical oxidants. Therefore, in our experiments, the concentration of micropollutants and organic matter in the membrane retentates were reduced by treatment with a perovskite thermocatalyst, namely, cerium-doped strontium ferrate (CSF), either during or after filtration. Bisphenol A (BPA) was chosen as a model pollutant to spark water samples, because it is a common water contaminant with a well-documented endocrine-disrupting activity and toxicity [18]. Moreover, common biological treatments are not effective for BPA degradation [19]. On the other hand, we reported full abatement of BPA in water by treatment with CSF perovskite in our previous study [20]. 

The purpose of this work was to integrate a ceramic membrane with perovskite in order to effectively retain and degrade BPA and improve the quality of feed and permeate. We also performed tests with real secondary effluent from treatment wastewater, which contain large amounts of non-toxic organic matter. Organic matter may have a negative influence on the thermocatalytic abatement of micropollutants and can cause clogging of membranes’ pores at the detriment of membrane permeance [21]. It is responsible for membrane fouling and for the chemical oxygen demand of the retentate. Therefore, the influence of the thermocatalyst on reduction of non-toxic organic matter content and fouling is also discussed. Finally, we performed a two-step experiment to check the impact of thermocatalyst on BPA abatement and organic matter degradation for effluent concentrate in order to compare it with one-step filtration with thermocatalyst addition.

## 2. Materials and Methods

### 2.1. Cerium-Doped Strontium Ferrate Perovskite Synthesis

Initially, 1.8 g strontium nitrate (Carl Roth, purity ≥ 99%)_,_ 4.04 g iron (III) nitrate (Sigma-Aldrich, purity ≥ 98%) and 0.65 g cerium (III) nitrate (Sigma-Aldrich, purity 99%) were completely dissolved in 200 mL of distillated water, using a 1 L glass beaker as container. Then, 7.68 g citric acid (Carl Roth, purity ≥ 99.5%) were added in order to reach a citric acid-to-metal cations ratio of 2, whereas the reducers-to-oxidizers ratio (Φ) was regulated at its stoichiometric value through the addition of 9.25 g of ammonium nitrate (Sigma-Aldrich, purity ≥ 99.5%), according to the valence concepts based on propellant chemistry [22]. The pH of the solution was adjusted to 6.0 using ammonium hydroxide (Sigma-Aldrich, 25 wt %), in order to favor citrate anions-metal cations complex formation, and the glass beaker was placed on a hot plate and kept under 80 °C for the evaporation of the water under continuous magnetic stirring. When a sticky gel was obtained, the hot plate was set to the maximum temperature (310 °C) in order to start the gel self-ignition. After the combustion, the as-burned powder was calcined in 1000 °C for 5 h with a heating rate of 5 °C min^−1^. After calcination, about 2 g of Sr_0.85_Ce_0.15_FeO_3−δ_ powder were obtained. 

### 2.2. Membrane Fabrication

The membrane preparation method is described in detail by Ma et al. [14]. Note that 5% mol Al_2_O_3_-doped silica NF membrane used for tests was fabricated via the sol-gel method using a cationic surfactant, namely, N,N,N-Trimethylhexadecan-1-aminium bromide (CTAB) as pore-forming agent. The molar ratio of surfactant/oxide was kept at 0.5, because, based on the previous study, the membrane prepared under such conditions exhibited the best selectivity towards organic pollutants and salts. A two-step approach was applied for the sol synthesis. The first step of synthesis was the hydrolysis of TEOS, which was achieved by letting to react a mixture of TEOS (98%, Sigma-Aldrich, St. Louis, MO, USA), ethanol (99.9%, VWR Chemicals, Radnor, PA, USA), deionized water, and nitric acid (69%, Sigma-Aldrich, St. Louis, MO, USA), with a molar ratio of 1:4:2.5:0.04, at 60 °C for 3 h. Then, in the second step of the synthesis, CTAB (99%, Sigma-Aldrich, St. Louis, MO, USA) was added to the pre-hydrolyzed TEOS solution to achieve the desired CTAB: (SiO_2_ + Al_2_O_3_) molar ratio. After the complete dissolution of CTAB, aluminum isopropoxide (AIP) (98%, Sigma-Aldrich, St. Louis, MO, USA) was directly added to the mixture to obtain a 5 mol% Al_2_O_3_ concentration in the final consolidated membrane material. The mixture was continuously stirred at 60 °C until all the AIP was dissolved and a transparent yellowish solution was obtained). Sol was diluted by 1:20 volume ratio with ethanol and subsequently filtered with a 0.2 µm syringe filter (Minisart RC, 25 mm, Sigma-Aldrich, St. Louis, MO, USA) to remove dust particles and impurities before the coating. The membrane was coated on commercial y-alumina tubular support with y-alumina intermedia layer (250 × 10 × 7 mm (L × OD × ID), Pervatech B.V., Rijssen, The Netherlands). The membrane was fabricated by dip-coating of the alumina-doped silica sols onto the supporting substrates. Specifically, the inside of the supports was coated vertical by a lab-made device at a dipping/withdrawing rate of <2.5 cm/min. After drying at room temperature for 24 h, the membranes were calcined at 450 °C for 2 h at the heating and cooling rate of 2 °C min^−1^.

### 2.3. SEM

The morphology of the membrane cross-section and surface was investigated by SEM analysis using an EVO 50 XVP microscope (Zeiss, Köln, Germany) with LaB6 source. The samples were mounted on metallic stubs with double-sided conductive tape and ion coated with a gold layer (thickness ~25 nm) by a sputter coater (Baltec SCD 050, Pfäffikon, Switzerland) for 60 s under vacuum at a current intensity of 60 mA to avoid any charging effect.

### 2.4. Effluent Sampling

Secondary effluent was sampled from the Wastewater Treatment Plant Aalborg West (AAW) 57.049422° N, 9.864735° E in Denmark in January 2021. Sampling permission was granted by Aalborg Forsyning, Kloak A/S. Samples were transported to the laboratory within 1 h after sampling and kept in the fridge until experiments. Samples were used for NF permeance tests without and with addition of thermocatalyst and for experiments with concentrate.

### 2.5. Nanofiltration Apparatus

**Figure 1:**
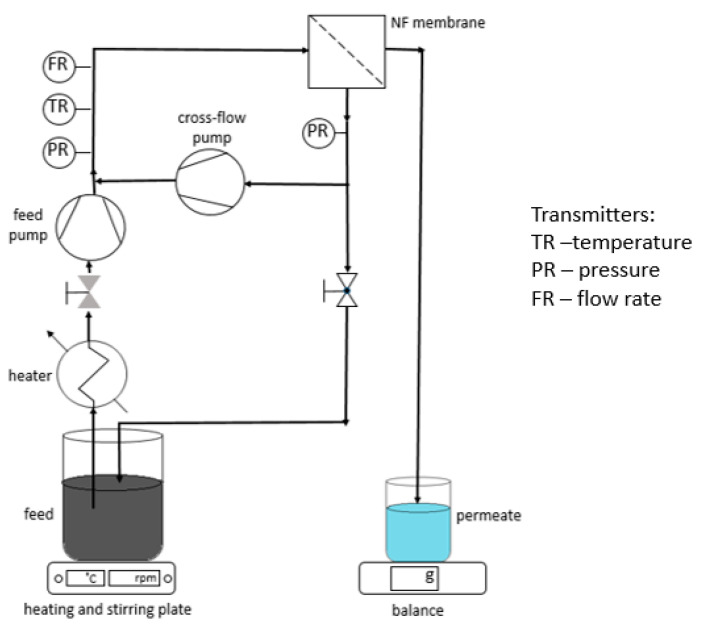
Scheme of the nanofiltration setup used in this study.

The experimental cross-flow filtration set-up is shown in Figure 1, and it follows the system described by Farsi et al. [23]. The feed was pumped to the NF membrane by a feed pump giving pressure 6 bar. Monotubular membrane (250 × 10 × 7 mm (L × OD × ID), Pervatech B.V., The Netherlands) was sealed completely in a stainless steel membrane module. The effective membrane surface was 55 cm^2^. The permeate mass flow was measured by a balance (Mettler Toledo, Mono Bloc series, Greifensee, Switzerland) connected to a computer to continuously log the weight of the permeate. The feed pressure was measured before and after the membrane by two pressure transmitters (Danfoss, MBS 4010, Nordborg, Denmark) and an electronic heat sensor (Kamstrup A/S, Skanderborg Denmark) measured feed temperature before membrane module. A rotary lobe pump (Philipp Hilge GmbH & Co, Novalobe, Germany) generated the cross-flow rate measured by a microprocessor-based flow rate transmitter (Siemens, MAG 50000, Munich, Germany). It was adjusted to 1.6 ± 1 m s^−1^ for all the experiments. The retentate stream was controlled by a manual valve. The system was operated for 3 h to ensure that the system was operated at steady-state condition. During filtration, samples were collected from each stream (feed, permeate) at various times to observe system changes during time. Filtration experiments were done at temperature 50 °C. A typical test started by filling up the feed tank with 1.8 L of solution and setting the system at the specified operational conditions. 

### 2.6. COD Analysis

COD (chemical oxygen demand) analysis was performed using COD kits (Hach^®^) 5–60 mg/L and 15–150 mL/L and Hach Lange DR3900 apparatus. Then, 2 mL of samples were added to each vial from the proper kit and placed in a heating block for 2 h at 148 °C. After cooling down to 20 °C, the vials were placed one after another in Hach Lange to measure COD in mg L^−1^. 

### 2.7. Determination of BPA Concentration in Water Samples

In each experiment, collected samples of feed were filtered using RC 0.45 µm syringe filters. Then, the liquid phases of feed and permeate were analyzed through HPLC with UV detection (Phenomenex, with a Kintex^®^ 5µm EVO C18 100 Å LC column (150 × 4.60 mm), mobile phase flow of 1 mL min^−1^ (acetonitrile/water = 60/40 *v*/*v*%), UV detector at 230 nm) in order to determine the concentration of the contaminant in the sample. A calibration curve was determined using several solutions of BPA in concentrations between 1 and 10 mg L^−1^.

## 3. Results

### 3.1. SEM of Membrane

**Figure 2:**
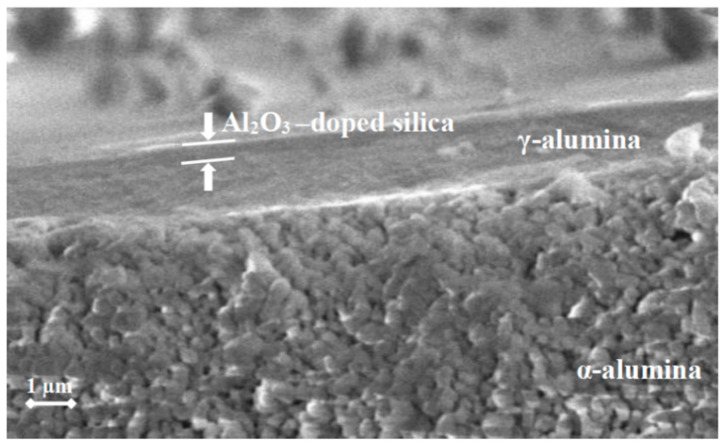
Cross-sectional SEM micrograph of the membrane used in this study. The Al_2_O_3_-doped silica NF layer is coated over a mesoporous γ-alumina interlayer and a macroporous α-alumina support.

Figure 2 shows the SEM cross-section of the Al_2_O_3_-doped silica membrane used in this study. The micrograph shows a continuous film covering the multilayered alumina support. From the picture, the thickness of the top layer measured to be 0.5 µm. Based on analysis at the low-temperature porosimeter [14], the Al_2_O_3_-doped silica material coated on the alumina support has main and maximum pore size of 1.3 nm and 2.5 nm, respectively, and therefore it is suitable to act as a NF membrane. Moreover, CSF particles have a flake-like structure with thickness of about 0.2 µm and lateral dimensions that are several micrometers large. Therefore, Al_2_O_3_-doped silica membrane can easily retain CSF particles at the feed side during filtration. 

### 3.2. BPA Rejection

**Figure 3:**
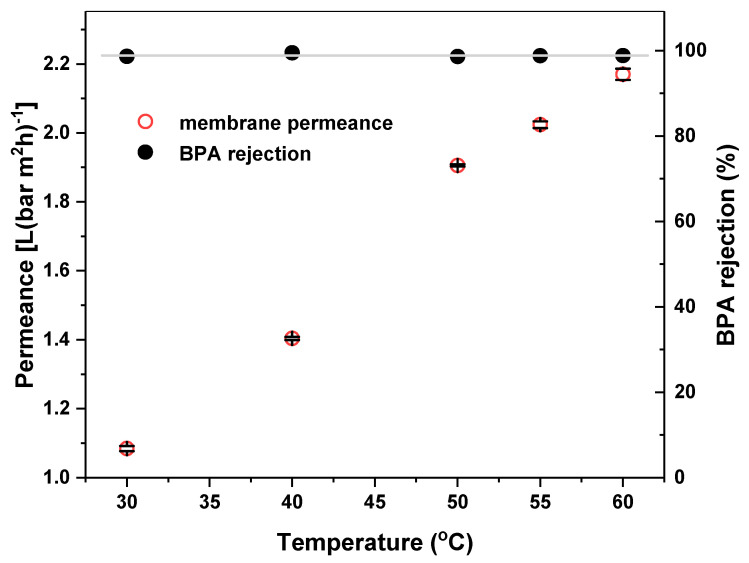
Impact of temperature on water permeance and BPA rejection for the Al_2_O_3_-doped silica NF membrane used in this study. Black bars indicate the error for the permeance values.

The experiment studying the BPA rejection and impact of temperature on water permeance was performed using 1.8 L of deionized water with a starting BPA concentration of 10 mg L^−1^ as membrane feed. The cross flow was set up to 1.6 ± 0.1 m s^−1^ and the feed was pumped with a trans-membrane pressure of 6 bar. From Figure 3, it can be seen that no impact of temperature on BPA rejection has been observed. Pollutant rejection remains constant, reaching values near to 100% at all the tested temperatures: 30 °C (98.7%), 40 °C (99.5%), 50 °C (98.6%), 55 °C (98.8%), 60 °C (98.9%). However, the water permeance of the membrane increased with increase of temperature in the range 30–60 °C as follows: 30 °C (1.09 L (h m^2^ bar)^−1^), 40 °C (1.40 L (h m^2^ bar)^−1^), 50 °C (1.91 L (h m^2^ bar)^−1^), 55 °C (2.02 L(h m^2^ bar)^−1^), 60 °C (2.17 L (h m^2^ bar)^−1^). This twofold increase of water permeance from 30 to 60 °C is consistent with the data reported by Tsuru et al. [24] for other types of ceramic NF membranes. 

### 3.3. BPA Abatement during Filtration

The experiments to investigate the thermocatalytic abatement during filtration were performed at a temperature of 50 °C, because our previous studies showed that CSF can catalyze fast degradation of BPA at this temperature [20]. The feed volume was 1.8 L, a cross flow of 1.6 ± 0.1 m s^−1^ was applied and the transmembrane pressure was 6 bar. The feed was heated and after reaching a temperature of 50 °C the proper amount of BPA was added to reach a concentration of pollutant of 10 mg L^−1^. After running the system for 2 h, the thermocatalyst was added in the retentate to reach a concentration of 1 g L^−1^ and the system was operated for another 3 h. 

**Figure 4:**
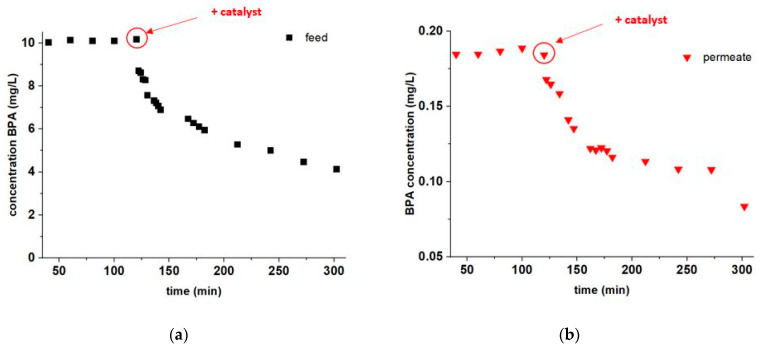
Degradation experiment at 50 °C with addition of CSF: (**a**) BPA concentration in the membrane feed as a function of the filtration time; (**b**) BPA concentration in the permeate as a function of the filtration time. Red arrows and circles indicate the time when CSF was added to the membrane feed solution.

In Figure 4a, it can be seen that during the first 2 h the concentration of BPA in the feed was stable at about 10 mg L^−1^, with a slight increase, which corresponds to the volumetric concentration factor (VCF), i.e., the ratio of the initial feed volume to the feed volume after a certain filtration time. Indeed, in our filtration experiment, VCF was only 1.069 after 2 h of filtration. Hence, the BPA concentrations in the feed during the first two hours indicate that this micropollutant is stable at the filtering temperature. On the contrary, after adding the CSF thermocatalyst, the concentration of BPA immediately decreases, reaching 4 g L^−1^ after 3 h of operation, despite a VCF = 1.241 for this filtration time. 

Figure 4b shows the development in concentration of BPA in the permeate over time. It can be seen that, during the first 2 h, the permeate concentration of BPA is stable, around 0.19 mg L^−1^, with a slight increase, which corresponds to the increase in concentration at the retentate side. After adding the thermocatalyst to the feed, the concentration of BPA in the permeate significantly decreases, reaching about 0.1 mg L^−1^ after 3 h. This experiment proves that the addition of thermocatalyst to the NF system leads not only to the abatement of BPA at the feed side, but it also improves the quality of the permeate. Indeed, the membrane selectivity remains constant at (98.1 ± 0.2)% during this filtration experiment (when excluding the outlying value measured for the permeate at 300 min). Therefore, the abatement of BPA concentration at the feed side, upon adding the CSF powder, corresponds to a decrease in BPA concentration at the permeate side. Moreover, the addition of CSF powder did not undermine BPA rejection, suggesting that CSF particles had not damaged the Al-doped silica NF layer (e.g., by friction) during the experiment. 

### 3.4. Fouling Mitigation

The experiments to investigate the influence of thermocatalyst on the reduction of non-toxic organic matter and fouling were conducted by filtering the secondary effluent collected from the Aalborg Wastewater Plant West (WWTP). The properties of effluent are listed in Table 1. 

**Figure 5:**
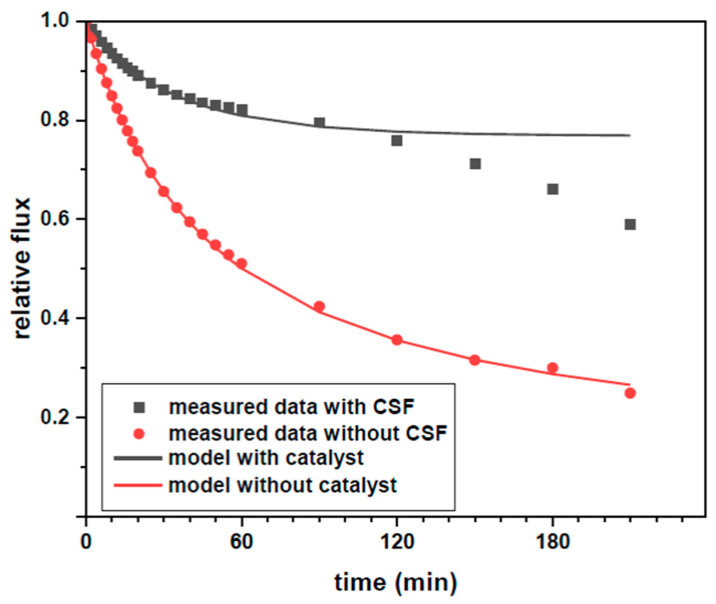
Comparison of change of relative flux in time for effluent with and without thermocatalyst; bullets indicate experimental points; lines correspond to an intermediate pore blocking model (Supplementary Materials).

As can be seen in Figure 5, when the NF membrane is used to filter a real wastewater effluent, the flux of the permeate decreases along the filtration time. The 80% of permeate flux decline can be explained by the membrane being fouled by the organic matter present in the wastewater effluent (COD 33.4 mg L^−1^). On the other hand, the flux decline of the permeate was only 20% when 1 g L^−1^ of CSF was added to the wastewater effluent during filtration. Moreover, the fouling was studied to determine which fouling type occurred during each experiment. A method based on a simple regression fitting [25] was used to determine the type of fouling mechanisms in experiments on the filtration with cross flow, as explained in detail in the Supplementary Materials. It was found that, for both the effluent with and without thermocatalyst, the main fouling type is intermediate pore blocking, for which the best (≈1) R^2^ correlations were found. It can be seen in Figure 5 that modelled data correspond well with the experimental data for filtration of effluent with (J_SS_ = 2.704 L m^−2^ h^−1^, K_i_ = 0.009) and without thermocatalyst (J_SS_ = 0.816 L m^−2^ h^−1^, K_i_ = 0.004). The intermediate pore blocking appeared to give slower fouling formation for experiments with thermocatalyst, which is explained by the lower content of organic matter to fouling the membrane as a result of organic matter degradation by the thermocatalyst. After about 100 min of filtration, the models deviate from intermediate pore blocking models, which may be a result of cake formation [25], which in this case can correspond to deposition of CSF particles on the membrane surface.

### 3.5. Thermocatalytic Treatment of the NF Concentrate

**Figure 6:**
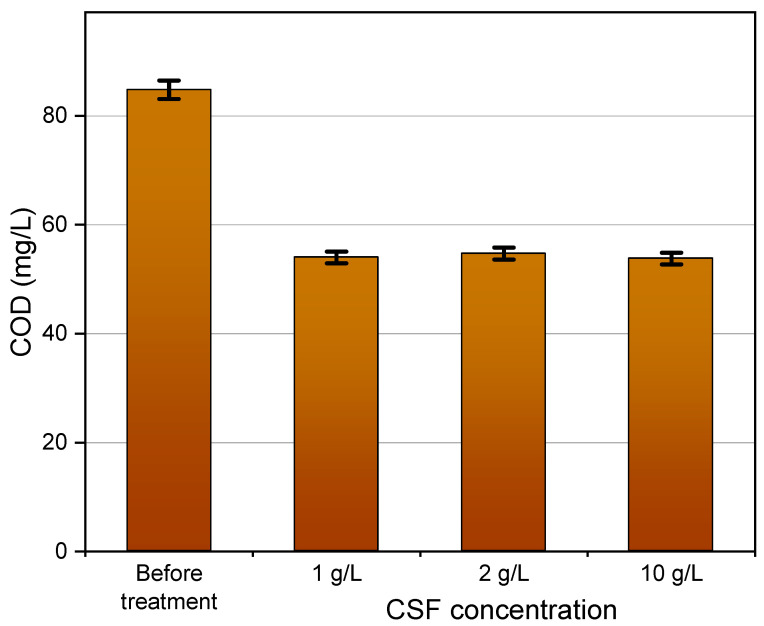
Chemical oxygen demand (COD) in the concentrated wastewater effluent before and after treatment with different amounts of CSF for 5 h.

**Figure 7:**
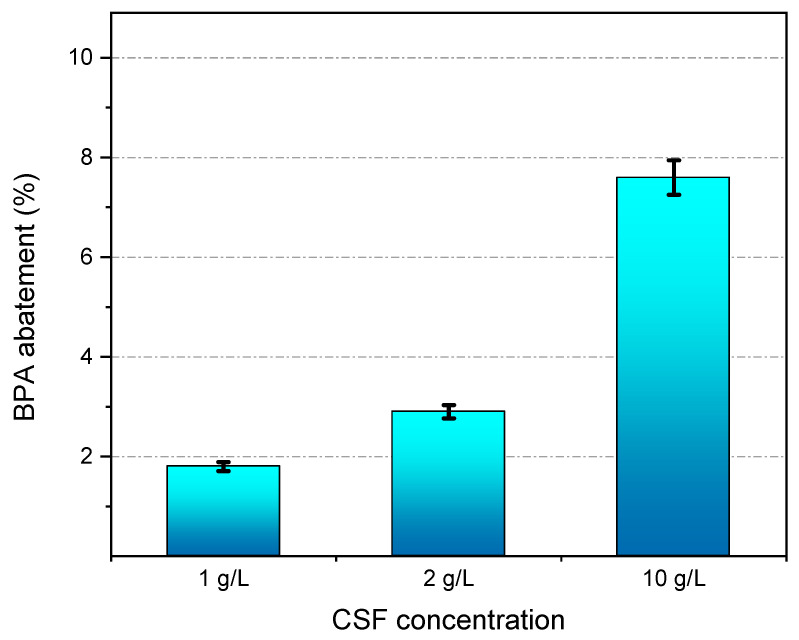
Abatement of BPA in the concentrated wastewater effluent after treatment with different amounts of CSF for 5 h.

A second way to integrate CSF with NF is to use thermocatalysis as a separate step after concentration, thus saving energy by reducing the volume of wastewater which needs to be heated. Therefore, we performed a new experiment in which 900 mL of wastewater effluent were concentrated to 180 mL by filtration at 6 bar over the Al_2_O_3_-doped silica membrane. The sample was sparked with BPA in order to reach a BPA concentration of ~10 mg L^−1^ after concentration. After concentration, 50 mL samples of the concentrate were treated at 50 °C with CSF at concentrations of 1, 2 and 10 g L^−1^ over 5 h. As can be seen in Figure 6, treatment with CSF thermocatalyst in a batch reactor causes 35% abatement of COD after 5 h of treatment. COD abatement does not change significantly by increasing CSF concentration from 1 to 10 g L^−1^. This result is consistent with the fact that the dissolved organic matter consists of different types of chemical species, some of which are highly recalcitrant to degradation, such as part of the humic substances. On the other hand, the abatement of BPA increases following the concentration of CSF, as shown in Figure 7. However, these tests show also that the thermocatalyst is less efficient in the abatement of BPA in real matrixes, which contain large quantities of dissolved organic matter, than when it was tested with model solutions of BPA dissolved in deionized water. 

## 4. Discussion

In this study, we presented a new method of water purification using the recently developed Al_2_O_3_-doped silica NF membrane combined with cerium-doped strontium ferrate (CSF), as thermocatalyst for the abatement of water pollutants. The new process was investigated in the degradation of bisphenol A (BPA), which is a common water contaminant with endocrine-disrupting activity. Concerning the NF membrane, we observed no impact of temperature on BPA rejection, which remains >98% at all the tested temperatures (30–60 °C). Instead, water permeance showed a twofold increment by increasing the feed temperature form 30 to 60 °C. Such temperature-permeance dependence in ceramic NF membranes can be explained by considering the change in solvent viscosity and that permeation in micropores occurs by a combination of viscous flow and activated transport [24]. Hence, the increase of the feed temperature is beneficial for membrane permeance and for the thermocatalytic abatement at the same time [20].

Two possible configurations were tested in this study, each of them with some specific advantages. Addition of CSF at the membrane feed during filtration allows for micropollutant abatement, while mitigating membrane fouling and improving the quality of the permeate. On the other hand, pre-concentration of the wastewater by nanofiltration allows for a strong reduction of the thermal energy needed for the thermocatalytic process, and decreases investment and running costs of the abatement step, since a smaller wastewater volume needs to be treated [26]. The two different configurations can be selected based on the type of wastewater, on its temperature, and on the presence of low-grade waste heat or renewable waste heat.

The experiments reported in this paper can also highlight some of the challenges for the implementation of this technology on a real scale. Firstly, non-toxic dissolved organic matter, which is typically present in wastewaters at concentrations much higher than the micropollutants, has a negative effect on both the water permeance of the membrane [27] and the thermocatalytic performances of CSF in the abatement of micropollutants. In this study, we show that CSF can degrade part of the dissolved organic matter and that, when added in the membrane feed, had also a positive impact on fouling. However, we also observed that CSF was able to degrade about 60% of BPA in deionized water after 3 h at 50 °C and less than 8% of BPA in concentrated wastewater (COD~85 mg L^−1^) after 5 h at 50 °C. Therefore, thermocatalyst and process parameters should be optimized, taking into account the presence of non-toxic organic matter in real wastewater systems. A second challenge is the process upscaling. In this work, CSF was synthetized in a few grams batches by the solution-combustion method, which is notoriously not amenable to scale up. Nevertheless, Deganello et al. have indicated some strategies for large-scale synthesis of perovskites [27] and the industrial production of CSF is one of the tasks of the project NanoPerWater (EUREKA, Eurostars Cut-off 12, Project number: 113625). For the sake of comparison, all the thermocatalytic tests in this work were performed with dispersed CSF powders. Nevertheless, the recovery and reuse of the thermocatalyst is also a crucial aspect for this technology; especially when CSF is used in a separate abatement step after NF pre-concentration, and thus it cannot be retained by a membrane. For this reason, a possible implementation of this technology consists in the immobilization of the catalyst in a fixed-bed reactor for the abatement of micropollutants from wastewater effluent after pre-concentration over a NF membrane, which is indeed the scope of the recently funded NanoTheC-Aba project (JPI, 1st Aquatic Pollutants Joint Call 2020, Project number: ID 402). Concerning the economy of the new process, Ma et al. [14] have calculated that the Al_2_O_3_-doped silica NF membrane can operate at a specific energy consumption <0.15 kWh per m^3^ of permeate, which makes this step potentially attractive when organic contaminants need to be removed from wastewater. Nevertheless, the thermocatalytic step requires at least 1.167 kWh (m^3^ °C)^−1^ for heating wastewater, making the overall process expensive, unless the wastewater stream to be treated already has a temperature suitable for CSF activation, or low-grade waste heat is available on site (which is the case for many industrial processes), or it is possible to exploit solar thermal energy. 

## 5. Conclusions

For the first time, a thermocatalytic perovskite, namely Ce-doped strontium ferrate (CSF), was combined with a NF ceramic membrane for the treatment of wastewater. We showed that the addition of CSF to the membrane feed causes degradation of BPA and reduces BPA traces in the permeate. When the system was tested with a real wastewater effluent, CSF was able to reduce membrane fouling. From analysis of flux over time using different fouling models, it was found that the main fouling type occurring in our experiments is intermediate pore blocking. Our data show also that CSF can effectively degrade part of the non-toxic organic matter present in the water, which can explain its ability to mitigate membrane fouling. CSF can be also used to reduce the COD of wastewater after concentration by NF, although its ability to degrade BPA, and presumably the other micropollutants, is reduced by the scavenging effect of large concentrations of non-toxic organic matter, which also interacts with the reactive oxygen species generated by the thermocatalyst. Despite the abovementioned challenges, the new technology does not require light sources or additions of chemicals, contrary to other hybrid NF-advanced oxidation processes, e.g., those based on photocatalysis or Fenton technologies, respectively. Hence, integration of NF with thermocatalysis has the potential to rise as a new strategy for the treatment of wastewaters contaminated by micropollutants.

## Figures and Tables

**Table 1:** Properties of effluent.

Parameter	Unit	Value
pH (22.0 °C)		7.48 ± 0.01
Conductivity (22.5 °C)	mS/cm	1.15 ± 0.01
COD	mg/L	33.4 ± 0.7

## Data Availability

Data are contained within the article or Supplementary Materials.

## Supplementary Materials

The following are available online at https://www.mdpi.com/article/10.3390/membranes11080639/s1: Paragraph S1: Analysis of fouling models; Table S1: Results of R2 for each fouling method for filtration with and without catalyst.

## References

[1] Valbonesi P., Pro M., Vasumini I., Fabbri E. (2021). Science of the Total Environment Contaminants of emerging concern in drinking water: Quality assessment by combining chemical and biological analysis. Sci. Total Environ..

[2] Shahkaramipour N., Tran T.N., Ramanan S., Lin H. (2017). Membranes with Surface-Enhanced Antifouling Properties for Water Purification. Membranes.

[3] Chen H., Ku J., Wang L. (2019). Thermal catalysis under dark ambient conditions in environmental remediation: Fundamental principles, development, and challenges. Chin. J. Catal..

[4] Werber J.R., Osuji C.O., Elimelech M. (2016). Materials for next-generation desalination and water purification membranes. Nat. Rev. Mater..

[5] Anderson M.G., Mcdonnell J., Ximing C., Cline S.A., Balance W.W., Rockstrom J., Daily G.C., Ehrlich P.R., Reidy C.A., Dynesius M. (2006). The Challenge of Micropollutants in Aquatic Systems. Science.

[6] Xu R., Qin W., Tian Z., He Y., Wang X., Wen X. (2020). Enhanced micropollutants removal by nanofiltration and their environmental risks in wastewater reclamation: A pilot-scale study. Sci. Total Environ..

[7] Escalona I., Fortuny A., Stüber F., Bengoa C., Fabregat A., Font J. (2014). Fenton coupled with nanofiltration for elimination of Bisphenol A. Desalination.

[8] Li C., Sun W., Lu Z., Ao X., Li S. (2020). Ceramic nanocomposite membranes and membrane fouling: A review. Water Res..

[9] Wang P., Wang F., Jiang H., Zhang Y., Zhao M., Xiong R., Ma J. (2020). Strong improvement of nanofiltration performance on micropollutant removal and reduction of membrane fouling by hydrolyzed-aluminum nanoparticles. Water Res..

[10] Abdel-Fatah M.A. (2018). Nanofiltration systems and applications in wastewater treatment: Review article. Ain Shams Eng. J..

[11] Farsi A., Malvache C., de Bartolis O., Magnacca G., Kristensen P.K., Christensen M.L., Boffa V. (2017). Design and fabrication of silica-based nanofiltration membranes for water desalination and detoxification. Microporous Mesoporous Mater..

[12] García N., Purcell-milton F., Gun Y.K. (2020). Recent progress and future prospects in development of advanced materials for nano fi ltration. Mater. Today Commun..

[13] Chon K., Cho J. (2016). Fouling behavior of dissolved organic matter in nanofiltration membranes from a pilot-scale drinking water treatment plant: An autopsy study. Chem. Eng. J..

[14] Ma X., Janowska K., Boffa V., Fabbri D., Magnacca G., Calza P., Yue Y. (2019). Surfactant-assisted fabrication of alumina-doped amorphous silica nanofiltration membranes with enhanced water purification performances. Nanomaterials.

[15] Tsuru T. (2001). Inorganic porous membranes for liquid phase separation. Sep. Purif. Methods.

[16] Li N., Wang X., Zhang H., Zhang Z., Ding J., Lu J. (2019). Comparing the performance of various nanofiltration membranes in advanced oxidation-nanofiltration treatment of reverse osmosis concentrates. Environ. Sci. Pollut. Res..

[17] Wang J.L., Xu L.E.J.I.N. (2012). Advanced Oxidation Processes for Wastewater Treatment: Formation of Hydroxyl Radical and Application Advanced Oxidation Processes for Wastewater Treatment: Formation of Hydroxyl Radical. Crit. Rev. Environ. Sci. Technol..

[18] Arca-Ramos A., Eibes G., Feijoo G., Lema J.M., Moreira M.T. (2015). Potentiality of a ceramic membrane reactor for the laccase-catalyzed removal of bisphenol A from secondary effluents. Appl. Microbiol. Biotechnol..

[19] Zielińska M., Cydzik-Kwiatkowska A., Bułkowska K., Bernat K., Wojnowska-Baryła I. (2017). Treatment of Bisphenol A-Containing Effluents from Aerobic Granular Sludge Reactors with the Use of Microfiltration and Ultrafiltration Ceramic Membranes. Water. Air Soil Pollut..

[20] Janowska K., Boffa V., Jørgensen M.K., Quist-Jensen C.A., Hubac F., Deganello F., Coelho F.E.B., Magnacca G. (2020). Thermocatalytic membrane distillation for clean water production. NPJ Clean Water.

[21] He Z., Lyu Z., Gu Q., Zhang L., Wang J. (2019). Ceramic-based membranes for water and wastewater treatment. Colloids Surfaces A Physicochem. Eng..

[22] Deganello F., Tyagi A.K. (2018). Solution combustion synthesis, energy and environment: Best parameters for better materials. Prog. Cryst. Growth Charact. Mater..

[23] Farsi A., Boffa V., Qureshi H.F., Nijmeijer A., Winnubst L., Christensen M.L. (2014). Modeling water flux and salt rejection of mesoporous γ-alumina and microporous organosilica membranes. J. Membr. Sci..

[24] Tsuru T., Izumi S., Yoshioka T., Asaeda M. (2000). Temperature Effect on Transport Performance by Inorganic Nanofiltration Membranes. AIChE J..

[25] Mora F., Karla P., Quezada C., Herrera C., Cassano A. (2019). Impact of Membrane Pore Size on the Clarification Performance of Grape Marc Extract by Microfiltration. Membranes.

[26] Miralles-Cuevas S., Oller I., Agüera A., Pérez J.A.S., Sánchez-Moreno R., Malato S. (2016). Is the combination of nanofiltration membranes and AOPs for removing microcontaminants cost effective in real municipal wastewater effluents?. Environ. Sci. Water Res. Technol..

[27] Oller I., Miralles-cuevas S., Agüera A., Malato S. (2018). Monitoring and Removal of Organic Micro-pollutants by Combining Membrane Technologies with Advanced Oxidation Processes. Curr. Org. Chem..

