# Supplementary material for: Combined Nanofiltration and Thermocatalysis for the Simultaneous Degradation of Micropollutants, Fouling Mitigation and Water Purification
Source: Membranes (Basel). 2021 Aug 19;11(8):639. doi: 10.3390/membranes11080639 (PMC8401960; doi:10.3390/membranes11080639)
Supplement: Supplementary file 1 [file membranes-11-00639-s001.zip › membranes-1325002-supplementary.pdf]

Combined nanofiltration and thermocatalysis for the simultaneous degradation of micropollutants, fouling mitigation and water purification

Katarzyna Janowska [1], Xianzheng Ma [1], Vittorio Boffa [1,*], Mads Koustrup Jørgensen[1] and Victor M. Candelario[2]

[1] Center for Membrane Technology, Department of Chemistry and Bioscience, Aalborg University,
Fredrik Bajers Vej 7H, 9220 Aalborg, Denmark; kaj@bio.aau.dk (K.J); .xm@bio.aau.dk (X.M.); mkj@bio.aau.dk (M.K.J.)

[2] Department of Research and Development, LiqTech Ceramics A/S, Industriparken 22C, 2750 Ballerup, Denmark; vcl@liqtech.com (V.M.C.)

* Correspondence: vb@bio.aau.dk; Tel.: +45-9940-3579

1. Analysis of fouling models

1.1. Calculation of flow change in time based on measured weight change in time using polynomial fit followed by differentiation

$$m_A(t) = k_6t^6 + k_5t^5 + \cdots + k_1t + k \tag{S1}$$

$$-\frac{dm_A}{dt} = -(6k_6t^5 + 5k_5t^4 + \cdots k_1 + 0) \tag{S2}$$

Based on change of weight in time the polynomial fits were found for both effluent with and without catalyst and the changes of flow in time were calculated for both.

1.2. Calculation of flux based on the flow

$$J = \frac{m(t)}{A} \tag{S3}$$

Where m(t) – flow, A- membrane area (m^2)

1.3. Calculation of flux for different fouling mechanisms during crossflow based on the mathematical models described by Mora et al. [1]

a) complete pore blocking, n=2

$$J = (J_0 - J_{SS})\exp(-K_Ct) + J_{SS} \tag{S4}$$

b) standard pore blocking, n=1.5

$$J = \left(J_0^{-0.5} + K_S t\right)^{-2} \quad \text{(S5)}$$

c) intermediate pore blocking, n=1

$$J = \frac{J_{SS}}{\left[1-\left(\frac{J_0-J_{SS}}{J_0}\right)\exp\left(-J_{SS}K_i t\right]\right.} \quad \text{(S6)}$$

d) cake formation, n=0

$$K_g t = \frac{1}{J_{SS}^2}\left[ln\left(\frac{J}{J_0} * \frac{J_0-J_{SS}}{J-J_{SS}}\right) - J_{SS} * \left(\frac{1}{J} - \frac{1}{J_0}\right)\right] \quad \text{(S7)}$$

First the parameters J_0, J_{SS} and K were found. J_0 was set as the first modelled flux result for t=0. Afterwards the values of J_{SS} and K were estimated in order to have the smallest error possible. This was done using the Solver function in Microsoft Office Excel. After that the fluxes for particular fouling methods were calculated and the difference between measured and modelled data for each fouling method was plotted.

1.4. Calculation of factor R2 for the best fouling fit

In order to find the fouling method that fits the best our experimental data correlation factor R2 was calculated as followed:

1.4.1. first the error between measured and modelled flux was calculated as a square root of the difference between measured and modelled flux:

$$error = (J - J_0)^2 \quad \text{(S8)}$$

1.4.2. SSR was calculated as the sum of error data for each fouling method:

$$SSR = sum(\sum error) \quad \text{(S9)}$$

1.4.3. Av was calculated as an average from modelled flux data:

$$Av = average\ (J) \quad \text{(S10)}$$

1.4.4. SS was calculated as a square root of the difference between modelled flux and Av:

$$SS = (J - Av)^2 \quad \text{(S11)}$$

1.4.5. SST was calculated as the sum of SS data for each fouling method:

$$SST = sum(\sum SS) \quad \text{(S12)}$$

1.4.6. Finally the R2 was calculated as follows:

$$R2 = 1 - \left(\frac{SSR}{SST}\right) \quad \text{(S13)}$$

Based on R2 result the best matching fouling method was chosen for the results the closest to 1. The best fit was found for intermediate pore blocking n=1 for both filtration with and without catalyst for which R2=0.99

Table S1. Results of R2 for each fouling method for filtration with and without catalyst.

**Filtration**	**With catalyst**				**Without catalyst**			
**n**	2	1.5	**1**	0.5	2	1.5	**1**	0.5
**R2**	0.987	0.695	**0.991**	0.987	0.994	0.967	**0.9994**	-2.47

References:

[1] F. Mora, P. Karla, C. Quezada, C. Herrera, and A. Cassano, "Impact of Membrane Pore Size on the Clarification Performance of Grape Marc Extract by Microfiltration," 2019.
